# Rapid Learning of Magnetic Compass Direction by C57BL/6 Mice in a 4-Armed ‘Plus’ Water Maze

**DOI:** 10.1371/journal.pone.0073112

**Published:** 2013-08-30

**Authors:** John B. Phillips, Paul W. Youmans, Rachel Muheim, Kelly A. Sloan, Lukas Landler, Michael S. Painter, Christopher R. Anderson

**Affiliations:** 1 Department of Biological Sciences, Virginia Tech, Blacksburg, Virginia, United States of America; 2 Department of Functional Zoology, Lund University, Lund, Sweden; 3 South Carolina Department of Natural Resources, Charleston, South Carolina, United States of America; 4 United States Naval Academy, Annapolis, Maryland, United States of America; University of Houston, United States of America

## Abstract

Magnetoreception has been demonstrated in all five vertebrate classes. In rodents, nest building experiments have shown the use of magnetic cues by two families of molerats, Siberian hamsters and C57BL/6 mice. However, assays widely used to study rodent spatial cognition (e.g. water maze, radial arm maze) have failed to provide evidence for the use of magnetic cues. Here we show that C57BL/6 mice can learn the magnetic direction of a submerged platform in a 4-armed (plus) water maze. Naïve mice were given two brief training trials. In each trial, a mouse was confined to one arm of the maze with the submerged platform at the outer end in a predetermined alignment relative to magnetic north. Between trials, the training arm and magnetic field were rotated by 180^°^ so that the mouse had to swim in the same magnetic direction to reach the submerged platform. The directional preference of each mouse was tested once in one of four magnetic field alignments by releasing it at the center of the maze with access to all four arms. Equal numbers of responses were obtained from mice tested in the four symmetrical magnetic field alignments. Findings show that two training trials are sufficient for mice to learn the magnetic direction of the submerged platform in a plus water maze. The success of these experiments may be explained by: (1) absence of alternative directional cues (2), rotation of magnetic field alignment, and (3) electromagnetic shielding to minimize radio frequency interference that has been shown to interfere with magnetic compass orientation of birds. These findings confirm that mice have a well-developed magnetic compass, and give further impetus to the question of whether epigeic rodents (e.g., mice and rats) have a photoreceptor-based magnetic compass similar to that found in amphibians and migratory birds.

## Introduction

Use of the geomagnetic field for spatial orientation has been documented in all five classes of vertebrates [1,2]. Among mammals, bats [3,4], two families of subterranean molerats [5–7], as well as Siberian hamsters and C57BL/6 mice [8–10], have been shown to rely on magnetic cues to determine compass headings. Magnetic cues have also been implicated in the tendency of free-living mammals as diverse as cattle, deer, and foxes to preferentially align their body axes along the North–South axis [11–13].

Evidence for learned magnetic compass orientation in Siberian hamsters and C57BL/6 mice is of particular interest because to date the large literature on rodent spatial behavior has yielded no other evidence for the use of magnetic cues in these, or other, epigeic rodents (i.e., species that are active above ground and, typically, have well-developed visual systems). It is noteworthy that all of the studies demonstrating the use of magnetic cues by rodents, with the exception of [14], have used assays that involve spontaneous or learned nest positioning behaviors [6,7,9,15]. Consequently, one possible explanation for the lack of evidence in other hamster and mouse studies is that rodents only utilize magnetic compass cues in certain behavioral contexts such as nest-building. However, given the obvious utility of a global reference system provided by the Earth’s magnetic field [1,16], the failure to use magnetic cues in other spatial tasks would be surprising, all the more so because magnetic responses in molerats appear quite robust, having been demonstrated in multiple species belonging to two different families and in studies carried out by at least four different laboratories [5–7,14,17,18].

Findings obtained over the last ~35 years indicate that there are two magnetoreception mechanisms in terrestrial vertebrates, based on fundamentally different biophysical processes [17,19–27].

Magnetite-based mechanisms involve single domain or interacting super-paramagnetic particles of biogenic magnetite believed to produce mechanical deformation of, or torque on, membrane structures that activate coupled membrane channels [28] or, in the case of freely-rotating single domain particles, to secondarily affect the rate of free-radical reactions that, in turn, influence the opening or closing of membrane channels [29]. Although in theory a magnetite-based mechanism could mediate responses that are sensitive to only the axis but not polarity, or to both the axis and polarity, of the magnetic field [28], to date all responses in which magnetite has been implicated are polarity sensitive [30–32].

A second class of mechanism is thought to involve a light-dependent biochemical reaction that forms long-lived, spin-correlated radical pair intermediates, i.e., the radical pair mechanism or RPM. To date, all of the responses in which a light-dependent, putatively radical pair-based mechanism has been implicated are sensitive to the axis, but not polarity, of the magnetic field [19,33], consistent with magnetic field effects on radical pair systems [34–36]. Animals with this type of ‘inclination’ compass use the slope or inclination of the magnetic field lines, instead of polarity (i.e., north vs. south), to distinguish between ’poleward’ and ’equatorward’ directions [33]. However, the most compelling evidence for a RP-based magnetic compass has come from behavioral experiments showing disruption of magnetic compass orientation by low-level radio frequency fields (1-10 MHz) that can alter the dependence of the radical pair reaction on magnetic field alignment [37–40].

Cryptochromes are the only animal photopigments known to form radical pair intermediates and are widely believed to play a role in the radical pair mechanism [34–36]. Absorption of a photon of light causes the flavin chromophore of cryptochrome to undergo an electron transfer reaction with an as yet unidentified electron donor resulting in the formation of the radical pair. The effect of an earth-strength magnetic field on the spin dynamics of the radical pair [34–36] is proposed to alter the formation or persistence of the cryptochrome’s signaling state, or of another downstream processes involved in phototransduction [34,36,38,39]. As a consequence, the response of photoreceptors containing an ordered array of light-activated cryptochrome molecules may show a complex dependence on magnetic field alignment [34,41,42].

Molerats are subterranean rodents adapted to live in aphotic habitats and exhibit spontaneous magnetic nest building responses that are consistent with a magnetite-based mechanism, i.e., independent of light, sensitive to the polarity of the magnetic field, and affected by brief magnetic pulses strong enough to remagnetize single domain particles of magnetite [5,17,43]. In contrast, the magnetoreception mechanism underlying learned nest building responses of C57BL/6 laboratory mice exhibits a complex pattern of response that is axially symmetrical (i.e., independent of polarity), consistent with the involvement of a radical pair mechanism [10] [16,21,25,33,39,44].

Reliance on magnetic cues may be especially important in novel surroundings, as shown in some species of migratory songbirds that use magnetic field cues for the initial calibration of star patterns [45]; but see 46. If epigeic rodents exposed to novel surroundings use the magnetic compass to ‘calibrate’ visual landmarks and then transfer control of spatial behavior from the magnetic reference to the visual landmarks, subsequent experimental manipulations such as rotation of visual landmarks, or changing the alignment of the magnetic field, will be unlikely to reveal the involvement of magnetic cues (see Discussion).

The present study was carried out to determine whether C57BL/6 mice: (1) can use magnetic cues to solve a water maze task carried out in an electromagnetically shielded enclosure that reduces levels of background radio frequency interference, and (2) can rapidly learn the relative position of a submerged platform with respect to the alignment of the magnetic field when they are first introduced to the novel surroundings. Experiments were carried out in a modified 4-arm (plus) water maze [47] enclosed within a pair of coils used to position an earth-strength magnetic field in one of four horizontal alignments coinciding with the four arms of the maze. To maximize the likelihood that mice would rely on magnetic cues, the testing apparatus was housed in a radially symmetrical room without directional visual, auditory, vibratory or olfactory cues.

## Materials and Methods

### Ethics Statement

Procedures used in these experiments were approved by the Virginia Tech Institutional Animal Care & Use Committee (IACUC) under DHHS Animal Welfare Assurance Number A3208-01.

### Behavioral Testing Facility

The Behavioral Testing Facility was designed for behavioral studies of magnetic field sensitivity. The facility consists of four testing buildings constructed of non-magnetic materials, with a central ‘hub’ building supplying air for heating and cooling, as well as filtered AC and DC power via underground conduits. Water maze experiments were housed in one of the testing buildings located 20 m from the hub building.

### Experimental Subjects

Subjects were female C57BL/6 mice that were 53-97 days old. Mice were originally purchased from Jackson Labs and then bred in our laboratory colony which was maintained on a L:D (15:9) light cycle. Males from the same colony were used in related nest-building experiments ([9] and unpubl. data). Groups of 6-8 mice were transported by car in group cages from the breeding colony to the Behavioral Testing Facility (~6 km) in the mid-morning (9:00-11:00), and placed in individual cages on a holding shelf outside the testing room. The light cycle in the holding room was the same as the breeding colony. In later tests, the holding shelf was shielded to minimize radio frequency interference (RFI); maximum signal strength < 0.10 nT (0.1-100 MHz).

### Water maze

**Figure 1:**
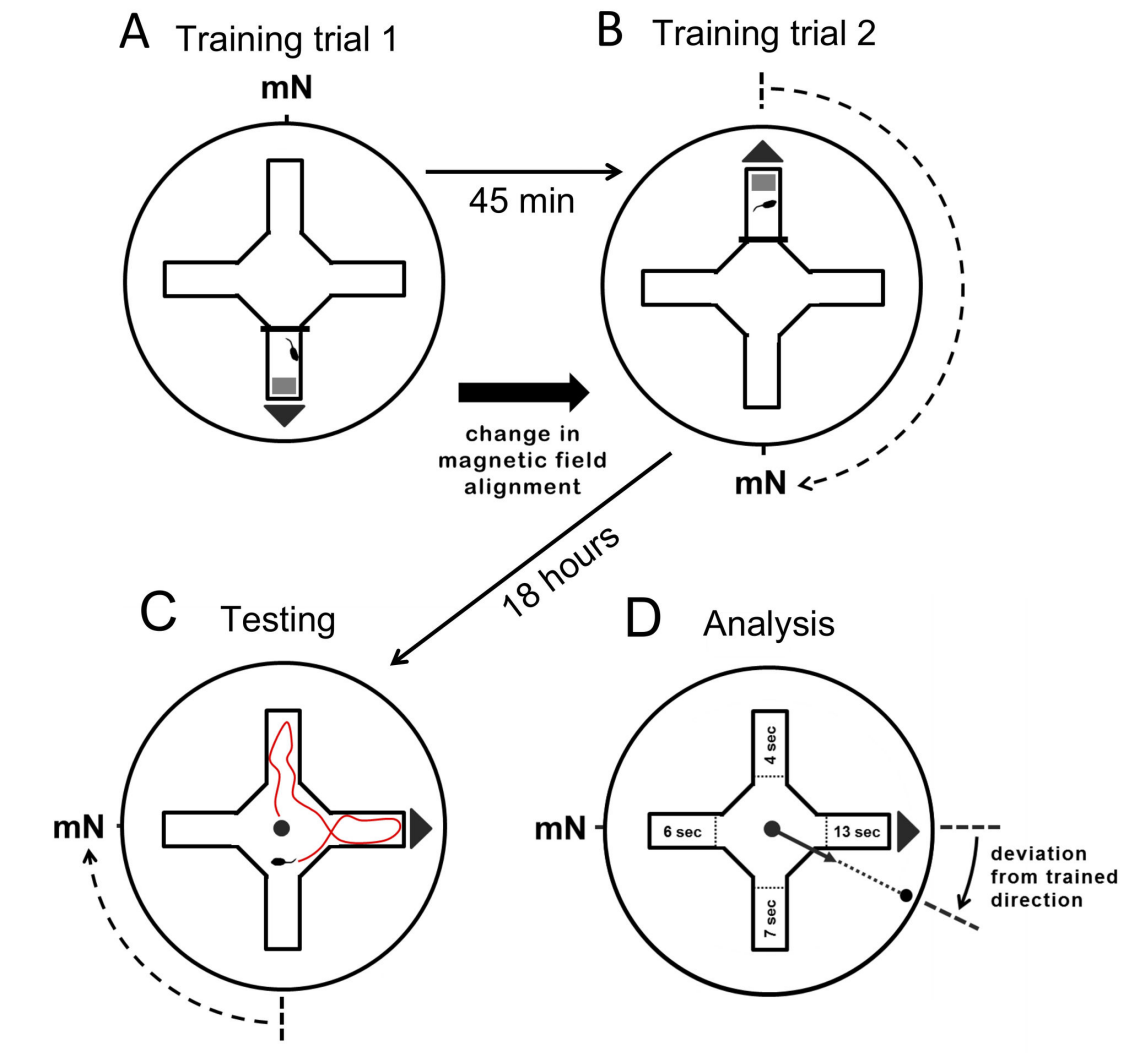
Training and testing protocol. Mice with no prior experience with the water maze were given two training trials in different arms of the water maze with the submerged platform in the same relative alignment to the magnetic field (A & B). In this example, the mouse is being trained to orient to magnetic south. For testing the following morning, the submerged platform was removed (C). The mice were released individually from a central release device (Figure S1) and had free access to all four arms of the maze. Magnetic field alignment was changed between trials, and data pooled across testing groups, so an equal number of mice were tested in each of the four magnetic field alignments, i.e., magnetic North (mN) at geographic North, East, South or West; each mouse tested only once. Orientation direction was calculated by the tracking software as the vector sum of the times spent in the four arms during the 60 sec testing trial (D).

**Figure 2:**
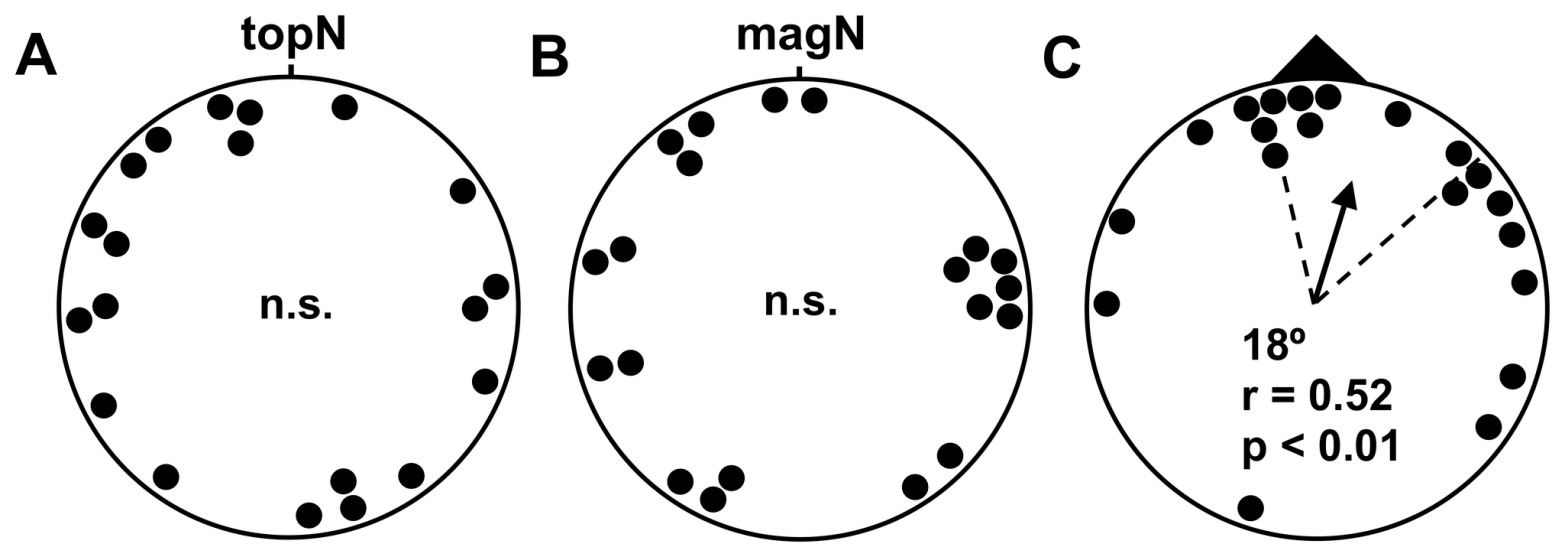
C57BL/6 mice rapidly learn the magnetic direction of a submerged platform in the plus water maze (data in Table S2). Directional responses from mice given two training trails in the late afternoon (Figure 1A & B), and then tested the following morning (Figure 1C). A) The distribution of topographic bearings, i.e., deviations from the north arm of the maze (topN), was indistinguishable from random (p > 0.10, Rayleigh test). B) The same was true of the distribution of magnetic bearings, i.e., deviations from the alignment of magnetic north in testing (magN). C) In contrast, the distribution of bearings relative to the trained magnetic direction (black triangle) was non-randomly distributed, and the 95% confidence interval for the mean vector bearing contained the trained direction. Each data point is the directional response of a single mouse tested in one of the four magnetic field alignments (see Methods & Methods). Arrow in the center of (C) is the mean vector for distributions of bearings that are non-randomly distributed. The length of the arrow is proportional to the mean vector length (r), a measure of the clustering of bearings ranging from 0 to 1; radius of the circle corresponds to r = 1. Dashed lines show the 95% confidence interval for the mean vector bearing [52]. ‘n.s.’- not significant (p > 0.10; Rayleigh Test).

**Figure 3:**
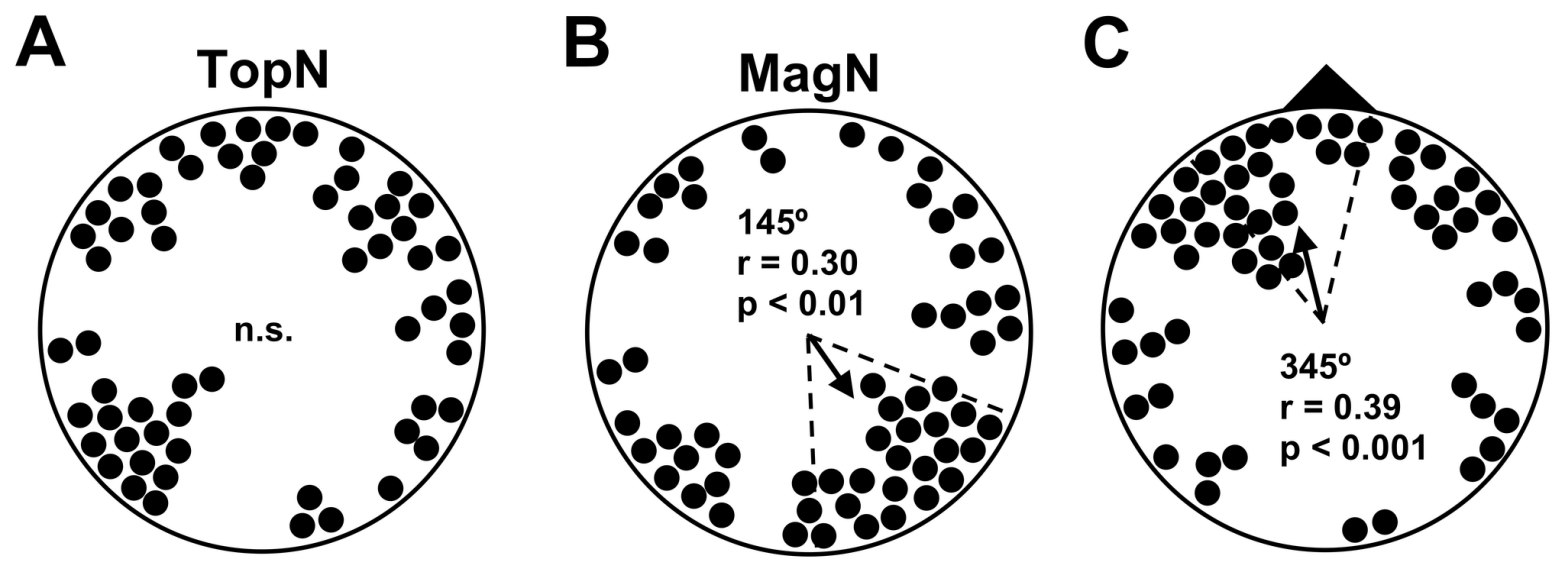
Replication of learned response (data in Table S3). A) The distribution of topographic bearings was indistinguishable from random (p > 0.10, Rayleigh test). B) The distributions of magnetic bearings from north- and south-trained mice were significantly different (p < 0.02, U^2^=0.253 Watson U^2^ test). Due to the unequal sample sizes (Table S3), however, these responses did not cancel out and the overall distribution of magnetic bearings was non-randomly distributed. C) The distribution of bearings relative to the trained magnetic direction (black triangle) was non-randomly distributed, and the 95% confidence interval for the mean vector bearing contained the trained direction.

Mice were trained and tested in a plus water maze ([47]; Figure 1), modified to include a centrally located release device (Figure S1). The plus maze was aligned so that the arms coincided with the cardinal compass directions. During training, a submerged 10.5 x 6.5 cm platform was located at the end of one of the four arms (~0.8 cm below the water surface). The water was made opaque with white tempera paint (Crayola Premier, non-toxic) so that the location of the submerged platform was not visible. Previous work has shown that water temperature has a strong effect on water maze performance [48]. In early experiments (Figure 2; Table S1) water temperatures were maintained between 28.5–30.0 °C, and in later experiments (Figure 3, Table S2) between 27.5–29.0 °C; at progressively higher water temperatures the trained response relative to the magnetic field deteriorated and then reversed (unpublished data). In both training and testing, the holding room was maintained at a temperature of 25.5-30 °C and the room containing the water maze was maintained at 27-31 °C and relative humidity of 60-90%.

The water maze was illuminated with white light from a ~1m diameter ring of rope lights (total length ~ 29 m consisting of ~1200 miniature bulbs; Utilitech Extra-Bright Clear Rope Light) coiled ~ 10 cm above a white 1.2 x 1.2 m Plexiglas diffuser. The rope lights were located above the electromagnetic shielding that enclosed the testing room. The video camera used to record the movements of the mice was also located above the electromagnetic shielding centered above a ~20 cm hole in the center of the diffuser that permitted an unobstructed view of the water surface. Light intensity at the water surface in the center of the plus maze was 28 lux.

### Magnetic fields

The water maze was centered in two horizontal, orthogonally aligned coils wrapped on the same cubical wooden frame [49]. The power supply connected to the coils (Lambda LQD-421) was located in the hub building, and connected to the coils by shielded wires running through an underground conduit. The wires were equipped with in-line EMI/RFI filters (Dearborn 1JX2459) that provided 62 dB of attenuation at 0.1 MHz and 80 dB of attenuation at > 1 MHz. The coils were positioned so that the horizontal component of the magnetic field could be aligned along the cardinal compass directions (magnetic north = geographic north, east, south or west [50]) coinciding with one of the four arms of the maze. The coils were double-wrapped, and controlled by reversing the direction of current flow in one of the two wraps [50,51]. Parallel current flow in one of the two double-wrapped Merritt et al. [49] coils produced a testing field with magnetic north aligned to either east or west, while parallel current flow in both coils produced a testing field with magnetic north aligned to south. Antiparallel current flow in both coils left the ambient field with magnetic north at north; for more information [9,50]. In all four testing field alignments, the current flow through the two wraps of wire remained the same, minimizing the possibility of non-magnetic artifacts associated with changes in magnetic field alignment. The three altered fields (magN = east, south, or west) closely resembled the ambient field (magN = north) in total intensity 51.0 µT ± 0.3 µT and inclination 64.5^°^ ± 1.5^°^.

### Design of the testing room to eliminate alternative (non-magnetic) cues

To maximize the likelihood that mice would use magnetic cues to solve a plus water maze task, the experimental chamber was designed to eliminate alternative sources of directional information. To minimize directional sound cues, the outbuilding containing the testing room was a sound insulated, triple walled structure, creating a building inside a building inside a building. To minimize visual asymmetries, the plus maze was centered in the 3m x 3m testing room, illuminated by a radially symmetric light centered overhead, and surrounded by a uniform white curtain. The temperature of the testing room was regulated by means of air circulated through underground ducts by fans located in the hub building. The air supply entered the testing room through a duct centered in the middle of the floor under the testing apparatus, and exited the room through a duct in the center of the ceiling, so that sound cues produced by the air supply, airflow, and any odor cues carried by the air were radially symmetrical. Sounds from the air supply also provided low-level white noise that helped to mask any remaining sounds (e.g., those caused by the observer quietly leaving the room after placing a mouse in the release device for a probe trial). Finally the water maze was supported on a vibration dampened concrete base to minimize directional vibrations carried by the substrate.

### Training

In the afternoon (15:00-17:00) of the same day the mice were transported to the Behavioral Testing Facility, they were given two brief training trials. An acrylic partition was used to restrict the movements of the mouse to one arm of the maze with the submerged platform at the outer end (Figure 1A & 1B). For each training trial, a mouse was removed from the holding shelf in its cage. The lid of the cage was removed, and the mouse was carried into the testing room in its holding cage. The mouse was grasped gently by the base of the tail, and placed into the training arm facing the center of the maze. The observer remained in the room until the training trail was over, standing in the same location relative to the water maze regardless of the magnetic field alignment and the arm of the maze in which the mouse was trained. Once a mouse reached the submerged platform, it would climb up on the platform and from there, up onto the side of the plus maze. The mouse was then given a delay of 10 sec before it was gently grasped by the tail, returned to its holding cage. During this 10 sec interval, some mice walked along the wall of the maze back toward the center of the maze, but this had no apparent effect on the learned response (data not shown). Any mouse that failed to climb up onto the platform and from there onto the wall of the maze within 60 sec in either training trial was excluded from the experiment.

Once the mouse was placed back in its cage, it was carried once around the water maze and then returned to the outer room. The cage was placed on a drying shelf beneath two 125 W infrared lights to dry the mouse’s fur. The back half of the drying shelf was shaded so the mouse could thermoregulate by moving between the front and back of the cage. After the fur of each mouse was dry (approximately 10 min), its cage was moved back to the holding shelf. Mice were given at least 45 min in their cages between each training trial to dry and rest, with water and food available *ad libitum*. After an interval of at least 45 min, each mouse was given a second training trial in the opposite arm of the maze. Prior to the second training trial, the alignment of the magnetic field was rotated by 180° so that magnetic north was in the same alignment relative to the training arm and to the submerged platform as in the initial training trial (Figure 1 middle diagram). As in the first training trial, the mouse was allowed to crawl up from the submerged platform onto the wall of the maze and remain there for 10 sec delay before being placed back in its cage and transported in the cage by an indirect route to the drying shelf in the outer room. In both training and testing, the holding room was maintained at a temperature of 25.5-30 °C and the room containing the water maze was maintained at 27-31 °C and relative humidity of 60-90%.

### Testing

Each mouse was given a single testing (probe) trial the following morning. For testing, the submerged platform and the partition used to isolate one arm of the maze for training were removed. The mouse was transferred from its holding cage to a light-tight cylindrical Plexiglas container (Figure S1). The cylinder was rotated slowly clockwise around its long axis as the mouse was carried into the testing room. The cylinder was then placed in the center of the arena from a constant direction (~45° relative to geomagnetic North) onto a round-topped acrylic rod in the center of the plus maze to serve as the release device. Once the release device was in place, the observer quietly left the room and closed the intervening doors. The release device was designed to gradually fill with water and, after ~50 sec, sink below the surface of the water releasing the mouse in the center of the water maze (Figure S1).

### Eliminating olfactory cues

In some preliminary experiments, members of the same litter showed similar absolute or ‘topographic’ responses, even when tested in different magnetic field alignments (Figure S3, Table S1). Consistent responses in the same absolute direction (i.e., independent of magnetic field alignment) are referred to as ‘topographic’, rather than ‘geographic’, because this type of response typically resulted from an asymmetry in the testing environment, e.g., directional sound cues audible inside the testing room or olfactory cues left by previous mice. To remove directional olfactory cues in the present experiments, the inside walls of the plus maze were scrubbed with a plastic sponge and the water from the four arms was thoroughly mixed between each probe trial. Although no evidence of ‘olfactory following’ was observed after this change in protocol, as a precaution littermates were tested in different magnetic field alignments so that any tendency of littermates to show similar topographic responses would decrease the consistency of orientation relative to magnetic north or relative to the trained magnetic direction.

### Data recording & analysis

Training and testing trials were recorded by a digital camera (6 frames/sec) centered above the air outflow in the ceiling of the testing room outside the electromagnetic shielding. A mouse’s path of movement during a testing trial was analyzed using video tracking software written by Rachel Muheim in Matlab version 7.14 (The MathWorks Inc., Natick, MA, USA). Each mouse’s position in the maze was recorded for 60 sec after it left the release area (defined by a circle of 12 cm radius centered on the release device). The tracking software provided a complete track of the mouse’s path, and calculated the amount of time (number of frames) the mouse spent in each of the four arms. The tracking program counted a mouse’s position as being in one of the arms, and therefore began to record the number of frames, when it crossed a line ~3 cm in from the entrance of the arm (~10% of the total arm length). Each mouse’s mean bearing was calculated by the tracking program as the vector sum of the time spent in the four arms. In preliminary experiments, initial arm entry was not found to be a reliable indicator of directional preference. This may have been due to the design of the release device (Figure S1). As the bottom section of the release device filled with water, the mouse would climb up and circle around the top rim (Figure S1D). Its initial swimming direction and arm entry was influenced by the direction the mouse happened to be facing when the bottom section sank.

Mice were tested in one of four magnetic field alignments (i.e., magnetic north = geomagnetic North, East, South, or West) coinciding with one of the four arms of the maze. Each mouse was tested only once, in one of the four fields. The direction of magnetic north was changed between trials, so that directional responses were obtained from an approximately equal number of mice tested in each of the four magnetic field alignments. Data are presented from the first 4 mice tested in each group, one in each alignment of the magnetic field. Directional responses were pooled across groups of mice. By pooling the responses as absolute or topographic bearings (ignoring the alignment of the magnetic field) and as magnetic bearings (deviations from the alignment of magnetic north in testing), the responses of mice could be partitioned into topographic and magnetic components. When approximately equal numbers of mice were trained to symmetrical directions (i.e., 2 directions separated by 180°, or 4 directions separated by 90°), the responses could also be partitioned into two magnetic components, i.e., a fixed preference relative to the magnetic field that was independent of training and a learned preference relative to the trained magnetic direction [9]. Mean bearings of individual mice (each tested only once) were treated as independent data. The distribution of mean bearings was analyzed for departure from a random distribution using the Rayleigh test (p < 0.05). Ninety-five percent confidence intervals around the significant group mean vectors were calculated to test for orientation with respect to the trained magnetic direction [52]. To determine if there were any residual effects of olfactory cues from littermates, the distribution of relative to the trained magnetic direction was separated into groups based on the number of littermates included in the test group (i.e., subgroups of 1-2 vs. 3+ littermates). The two distributions of bearings were compared using the Watson U^2^ test [52].

### Data excluded from analysis

Despite the efforts to isolate the testing room from directional sound cues, low frequency sounds penetrated the tripled walled structure (e.g., thunder, engine noise from farm and construction equipment). Based on criteria established prior to these experiments, data from a mouse were discarded when any potential source of directional sound cues was audible to the observer during a training or testing trial. Based on preliminary experiments, data were also discarded from individual mice for the following reasons: (1) the mouse grabbed the side wall while being placed in the maze for a training trial, (2) the mouse tried to climb the central post of the release device in a testing trial (Figure S1), or (3) the holding cage bumped into the ‘vestibule’ (Figure S2) as it was removed from the holding shelf for a training or testing trial (see Tables S2 and S3).

## Results

In the first test series, six groups of mice were trained to symmetrical directions relative to the magnetic field, i.e., trained direction = north (1 group), = south (1), = west (2), = east (2), and each mouse was tested once in only one of the four magnetic field alignments (magnetic north aligned to geomagnetic North, East, South, or West; see Materials & Methods). As in earlier nest-building experiments [9], this resulted in a total of 16 experimental conditions, i.e., 4 trained directions relative to the magnetic field x 4 alignments of the magnetic field in testing. Both the distribution of topographic bearings (i.e., absolute bearings, ignoring the alignment of the magnetic field in testing; Figure 2A) and the distribution of magnetic bearings (i.e., deviations of bearings from magnetic north, ignoring the trained direction; Figure 2B) were indistinguishable from random. In contrast, the distribution of bearings in Figure 2C was clustered in the trained magnetic direction (Table S2).

A similar response was obtained with a larger sample of mice trained to the north and south (Figure 3; Table S3). Evidence from preliminary experiments suggested that female mice may be able to recognize and follow odors left by their littermates in the plus water maze (Figure S3; Table S1). In the present experiments, ‘olfactory following’ was eliminated by scrubbing the inside walls of the maze and thoroughly mixing the water from the four arms between testing trials (see Materials & Methods). However, subsequent analyses of data pooled from both series of experiments (Figures 2 & 3) indicated that the remaining non-directional olfactory cues from littermates in the same testing group (i.e., the group of mice trained and tested together) may have influenced female behavior. The distribution of bearings relative to the trained magnetic direction from mice tested in littermate groups of 1 or 2 showed significantly less scatter than that from mice in littermate groups of 3 or more (Figure S4; p < 0.05, Watson U^2^ test). Ongoing research has confirmed the testing groups made up of subgroups of 1-2 littermates show strong magnetic compass responses comparable to those shown in Figure S4A.

## Discussion

The current findings show that C57BL/6 mice can use magnetic cues to solve a 4-armed (plus) water maze task. Given the large number of studies using water maze assays, it is surprising that earlier studies have not reported evidence for the use of magnetic cues. There are several possible reasons why previous studies have failed to find evidence for the use of magnetic cues. Magnetic cues may only be used when mice are presented with a task that favors the use of directional, rather than spatial, information, as is the case in the plus water maze. Magnetic cues may have less salience when alternative, non-magnetic cues such as visual landmarks (including the observer) are available or, more generally, when the problem or task the animal is confronting is more readily solved using local rather than global cues. Alternatively, mice may only attend to magnetic cues when first exposed to novel non-magnetic directional cues (i.e., unfamiliar landmarks) or surroundings.

Studies of migratory birds have shown that the Earth’s magnetic field can be used both as a primary source of compass information, and as a calibration reference for other directional cues, i.e., the sun and star compasses [1]. In nocturnal migrants, the magnetic compass is used to calibrate star patterns, but the ‘star compass’ then assumes primary control of migratory orientation even with the magnetic field present [45,46]. A similar phenomenon could occur in epigeic rodents if visual landmarks are initially calibrated relative to the magnetic field, but then take over primary control of directional and spatial behavior. For example, in water maze experiments, mice are often given acclimation trials prior to the onset of training to familiarize them with the task [53,54]. Any involvement of magnetic cues in the initial encoding (‘‘calibration’’) of non-magnetic spatial/directional cues may be missed unless manipulation of the relationship between magnetic and non-magnetic cues occurs when experimental subjects are first introduced into the water maze, as occurred in the present experiments. Further research is needed to determine if any of these factors, as well as background levels of RFI (see below), affect the use and relative importance of magnetic cues.

Despite these unresolved issues, there are several important conclusions to be drawn from the present study:

1 The findings confirm the results of earlier experiments showing that C57BL/6 mice, and in all likelihood other epigeic rodents, have a well-developed magnetic compass [8,9].
2 Use of magnetic cues is not limited to nest-building behavior [9]. Given evidence presented here for the use of magnetic compass cues in a water maze assay (Figures 2, 3), it is likely that magnetic cues will be found to be involved in other directional/spatial behaviors.
3 Mice can learn the magnetic direction of an important feature of the environment very quickly. Mice required only two brief (< 60 sec) training trials to learn the compass direction of the submerged platform in the plus water maze.

In the present experiments, stable magnetic compass responses were only observed when experiments were carried out in isolated buildings where ambient radio frequency fields were relatively low; the testing room was equipped with a single layer of grounded aluminum window screen, and electrical lines entering the shielded enclosure were equipped with in-line RFI/EMI filters that reduced levels of radio frequency interference in the water maze to < 0.1 nT at frequencies from 0.2 to 200 MHz (see also Figure S2). In the absence of such precautions, magnetic compass responses of mice in the water maze experiments, as well as mice and hamsters in earlier nest building experiments [8,9], varied in both strength and direction (Phillips unpubl. data). These findings suggest that sensitivity to low-level radio frequency fields, at intensity levels found in typical laboratory environments, may be a source of unexplained variability in studies of spatial behavior in animals that rely on radical pair-based magnetic compass.

Whether or not use of magnetic compass cues requires a low-RFI environment [55], understanding the role(s) of these cues in rodent spatial behavior and cognition is clearly of first importance. In contrast to visual, olfactory, auditory or vibrational ‘landmarks’, the geomagnetic field provides a global reference frame that can be used to help organize local landmark arrays [56] into a global map of familiar space. Use of such a global reference contrasts with the prevailing view that epigeic rodents use inertial guidance, and other ideothetic cues, to place visually isolated regions of the environment into register [57–60]. Consistent with this view, lesions of the vestibular nuclei have been shown to alter or eliminate directional responses at both behavioral and neural levels when rats move from familiar to unfamiliar surroundings [61–63]. In birds, however, neurophysiological responses have been recorded from the vestibular nuclei that are sensitive to both magnetic and gravitational input, suggesting that this may be a site where the integration of magnetic and gravitational information necessary for an inclination compass occurs [64]. Recent evidence from birds also suggests that the vestibular nuclei receives input from a magnetite-based receptor in the lagena [65,66] but see 67, or inner ear [67]. It remains to be determined if lesions of the vestibular nuclei in rodents eliminate input from an inclination magnetic compass or prevent integration of magnetic cues with other egocentric and allocentric cues used for path integration [68,69]. In the latter case, simple directional information provided by either a magnetite-based or radical pair-based mechanism would suffice [28].

If the magnetic compass of epigeic rodents is mediated by a radical pair mechanism, as appears to be the case in amphibians and birds [21,25,34,38,39,70,71], magnetic cues could play a variety of previously unforeseen roles in rodent spatial behavior and cognition. When photo-magnetoreceptors mediating a radical pair-based magnetic compass are located in the retina, as may be the case in birds and epigeic rodents [15,72–75], the magnetic field may be perceived as a complex, 3-dimensional pattern of light intensity or color superimposed on the animal’s surroundings and fixed in alignment with respect to the magnetic field [34,36,41]. The type of pattern produced by a radical pair mechanism could provide not only a global source of directional information, but also a simple spherical grid or coordinate system that could help to integrate spatial information from multiple sensory modalities [55].

In experiments with rats carried out in total darkness in an unshielded laboratory environment, Tryon et al. [76] found no evidence for magnetic field effects on the response of head direction cells, or the associated behavior, both during spontaneous activity and during attempts to condition the animals to directional magnetic stimuli. Unlike subterranean molerats [17,18], there is no evidence that epigeic rodents derive compass information from a light-independent, magnetite-based mechanism, although the involvement of this type of mechanism in a ‘map’ or geographic position sense is also a possibility.

Sensitivity to low-level radio frequency fields is a diagnostic property of the radical pair mechanism [34,37–39,42]. Studies of birds and cockroaches suggest that responses to earth-strength (~50 µT) magnetic fields can be disrupted by radio frequency fields tuned to the Larmor Frequency (the precession frequency of the electron spin dipole in the ambient magnetic field) at intensities as low as 10-15 nT [38,39,77,78]. Evidence that magnetic compass orientation in Siberian hamsters and C57BL/6 mice can be disrupted by background levels of radio frequency interference present in laboratory settings suggests that thresholds could be even lower (i.e., < 5 nT), although this level of sensitivity is difficult to reconcile with current theory [36,79].

## Conclusion

C57BL/6 mice have a well-developed magnetic compass sense that they readily use in nest-building [9] and water-maze (present study) tasks, both of which are learned responses. Given the value of the magnetic field as a global reference, it is difficult to imagine any directional or spatial behavior in which magnetic cues would not play at least some role, provided that the conditions necessary for detection of the magnetic field are met. The type of biophysical process that underlies the magnetic sense (i.e., either magnetite-based, or radical pair-based) may determine both the conditions under which the magnetic compass can operate, and the potential role(s) of magnetic cues in spatial behavior and cognition. Determining whether the magnetic compass of epigeic rodents is sensitive to low-level radio frequency fields and, if so, what the threshold is for such effects, has the potential to reveal fundamental properties underlying the detection and use of the geomagnetic field for spatial orientation.

## Supporting Information

Figure S1 **Water maze release device.** A) Mouse was transported from the holding shelf to the testing arena inside the opaque release device. Disks of paper towel attached with a ring cut from a self-adhesive label covered openings in both the top and bottom of the chamber. B) The release device was then slid slowly down onto a vertical Plexiglas rod extending up above the water in the center of the plus maze. The top of the rod inserted firmly into a socket centered on the bottom of the lid. C) The buoyancy of the lower section of the release device and the rate of inflow of water through four small openings were adjusted so that once the release device was in place, the lower section remained snugly up against the lid long enough for the observer to quietly exit the testing room and close the intervening door without being observed. D) Water slowly entered the lower chamber, and eventually the upper chamber, through the 4 small holes, causing the lower section of the release device to gradually separate from the lid and sink lower in the water. As the lower section filled with water and separated from the lid, the mouse invariably crawled up onto the top rim of the cylinder and spent 20-35 sec walking around the rim looking in all directions. E) The lower section of the release device gradually submerged, taking 45-50 sec for the top rim to sink below the surface, forcing the mouse to swim.
(TIFF)

Figure S2 **Holding shelf.** Mice were held in individual cages on non-magnetic shelves outside the testing room between training and testing trials. After returning from the water maze, mice were placed under a 75 W heat lamp that illuminated half the cage until their fur was dry. They were then returned to the holding shelves. Grounded shielding, consisting of two layers of aluminum window screen and an aluminum vestibule, was added between experimental series (Figures 2 and 3) to reduce ambient levels of radio frequency (RF) noise. Gray shading indicates RF intensities of < 0.1 nT after installation of shielding.
(TIFF)

Figure S3 **Evidence for olfactory following.** Example of data obtained in some tests before a cleaning protocol was introduced between testing trials to disperse directional olfactory cues left by the previous mouse. Distributions of bearings in A & B show the deviations between the topographic bearings of pairs of mice tested sequentially that were either littermates (A), or non-littermates (B); black arrow indicates the bearing of the first mouse of the pair. Although the alignment of the magnetic field was rotated by 90° between trials, A) the topographic bearings of 4 of 5 mice tested immediately after a littermate exhibited a topographic bearing that differed from that of the previous mouse by less than 20°. In contrast, B) the topographic bearings of the 4 mice tested immediately after a non-littermate showed no evidence of clustering. C) The deviations of the magnetic bearings of all 10 mice relative to the trained magnetic direction were non-randomly distributed, and the 95% confidence intervals included the direction opposite the trained direction (solid symbols—bearings from mice tested after a non-littermate, open symbols—bearings from mice tested after a littermate). The water temperature in testing was 31 °C, which causes a reversal in the direction of orientation relative to the trained magnetic direction (see Materials & Methods).
(TIFF)

Figure S4 **Number of littermates in the same testing group may affect the consistency of orientation.** Although mice from littermate groups of both (A) 1-2 and (B) 3+ exhibited significant orientation in the trained magnetic direction (p < 0.01, Rayleigh test), mice in littermate groups of 1-2 to exhibited less scatter (p < 0.05, Watson U^2^ test). Each mouse was trained in one of four directions (submerged platform towards magnetic north, east, south or west), and then tested in one of four alignments of the magnetic field (magnetic north aligned towards geomagnetic North, East, South, or West). Bearings are plotted relative to trained magnetic direction (black triangle at the top of each diagram). Arrows at the center of each distribution show the mean vector bearing; the length of the arrow is proportional to the mean vector length (‘r’) with the radius of the circle corresponding to r = 1. Dashed lines show the 95% C.I. for the mean vector bearing.
(TIFF)

Table S1 Responses of south trained mice in Figure S3.
(DOCX)

Table S2 Responses included in Figure 2 ^†^.
(DOCX)

Table S3 **Responses included in Figure 3^††^.**
(DOCX)

## References

[1] Wiltschko W, Wiltschko R (2005) Magnetic orientation and magnetoreception in birds and other animals. J Comp Physiol A Neuroethol Sens Neural Behav Physiol 191: 675-693. doi:10.1007/s00359-005-0627-7. PubMed: 15886990. 15886990 10.1007/s00359-005-0627-7

[2] Wiltschko R, Wiltschko W (1995) Magnetic orientation in animals. Berlin: Springer Verlag.

[3] Holland RA, Thorup K, Vonhof MJ, Cochran WW, Wikelski M (2006) Navigation: bat orientation using Earth’s magnetic field. Nature 444: 702. doi:10.1038/444702a. PubMed: 17151656. 17151656 10.1038/444702a

[4] Holland RA, Kirschvink JL, Doak TG, Wikelski M (2008) Bats use magnetite to detect the Earth’s magnetic field. PLOS ONE 3: e1676 PubMed: 18301753. 18301753 10.1371/journal.pone.0001676 PMC2246016

[5] Burda H, Marhold S, Westenberger T, Wiltschko R, Wiltschko W (1990) Magnetic compass orientation in the subterranean rodent *Cryptomys hottentotus* (Bathyergidae). Cell Mol Life Sci 46: 528-530. doi:10.1007/BF01954256. PubMed: 2347407. 10.1007/BF01954256 2347407

[6] Kimchi T, Terkel J (2001) Magnetic compass orientation in the blind mole rat *Spalax ehrenbergi* . J Exp Biol 204: 751-758. PubMed: 11171357. 11171357 10.1242/jeb.204.4.751

[7] Oliveriusová L, Nĕmec P, Králová Z, Sedláček F (2012) Magnetic compass orientation in two strictly subterranean rodents: learned or species-specific innate directional preference? J Exp Biol 215: 3649-3654. doi:10.1242/jeb.069625. PubMed: 22855619. 22855619 10.1242/jeb.069625

[8] Deutschlander ME, Freake MJ, Borland SC, Phillips JB, Madden RC, Anderson LE et al (2003) Learned magnetic compass orientation by the Siberian hamster, *Phodopus sungorus*. Anim Behav 65: 779-786 doi:10.1006/anbe.2003.2111.

[9] Muheim R, Edgar NM, Sloan KA, Phillips JB (2006) Magnetic compass orientation in C57BL/6J mice. Learn Behav 34: 366-373. doi:10.3758/BF03193201. PubMed: 17330527. 17330527 10.3758/bf03193201

[10] Painter MS, Dommer DH, Altizer WW, Muheim R, Phillips JB (2013) Spontaneous magnetic orientation in larval *Drosophila* shares properties with learned magnetic compass responses in adult flies and mice. J Exp Biol 216: 1307-1316. doi:10.1242/jeb.077404. PubMed: 23239891. 23239891 10.1242/jeb.077404

[11] Begall S, Cerveny J, Neef J, Vojtech O, Burda H (2008) Magnetic alignment in grazing and resting cattle and deer. Proc Natl Acad Sci USA 105: 13451-13455. doi:10.1073/pnas.0803650105. PubMed: 18725629. 18725629 10.1073/pnas.0803650105 PMC2533210

[12] Burda H, Begall S, Červený J, Neef J, Nemec P (2009) Extremely low-frequency electromagnetic fields disrupt magnetic alignment of ruminants. Proc Natl Acad Sci USA 106: 5708–5713. doi:10.1073/pnas.0811194106. PubMed: 19299504. 19299504 10.1073/pnas.0811194106 PMC2667019

[13] Červený J, Begall S, Koubek P, Nováková P, Burda H (2011) Directional preference may enhance hunting accuracy in foraging foxes. Biol Lett 7: 355-357. doi:10.1098/rsbl.2010.1145. PubMed: 21227977. 21227977 10.1098/rsbl.2010.1145 PMC3097881

[14] Kimchi T, Etienne AS, Terkel J (2004) A subterranean mammal uses the magnetic compass for path integration. Proc Natl Acad Sci USA 101: 1105–1109. doi:10.1073/pnas.0307560100. PubMed: 14732687. 14732687 10.1073/pnas.0307560100 PMC327158

[15] Olcese J, Reuss S, Semm P (1988) Geomagnetic field detection in rodents. Life Sci 42: 605-613. doi:10.1016/0024-3205(88)90451-1. PubMed: 3276998. 3276998 10.1016/0024-3205(88)90451-1

[16] Phillips JB, Jorge PE, Muheim R (2010) Light-dependent magnetic compass orientation in amphibians and insects: candidate receptors and candidate molecular mechanisms. J R Soc Interface 7 Suppl 2: S241-S256. doi:10.1098/rsif.2009.0459.focus. PubMed: 20124357. 20124357 10.1098/rsif.2009.0459.focus PMC2843995

[17] Marhold S, Wiltschko W, Burda H (1997) A magnetic polarity compass for direction finding in a subterranean mammal. Naturwissenschaften 84: 421-423. doi:10.1007/s001140050422.

[18] Thalau P, Ritz T, Burda H, Wegner RE, Wiltschko R (2006) The magnetic compass mechanisms of birds and rodents are based on different physical principles. J R Soc Interface 3: 583-587. doi:10.1098/rsif.2006.0130. PubMed: 16849254. 16849254 10.1098/rsif.2006.0130 PMC1664646

[19] Phillips JB (1986) Two magnetoreception pathways in a migratory salamander. Science 233: 765-767. doi:10.1126/science.3738508. PubMed: 3738508. 3738508 10.1126/science.3738508

[20] Wiltschko W, Munro U, Wiltschko R, Kirschvink JL (2002) Magnetite-based magnetoreception in birds: the effect of a biasing field and a pulse on migratory behavior. J Exp Biol 205: 3031–3037. PubMed: 12200406. 12200406 10.1242/jeb.205.19.3031

[21] Phillips JB, Borland SC (1992) Behavioural evidence for use of a light-dependent magnetoreception mechanism by a vertebrate. Nature 359: 142-144. doi:10.1038/359142a0.

[22] Phillips JB, Borland S (1994) Use of a specialized magnetoreception system for homing by the eastern red-spotted newt Notophthalmus viridescens. J Exp Biol 188: 275–291. PubMed: 9317797. 9317797 10.1242/jeb.188.1.275

[23] Beason R, Semm P (1996) Does the avian ophthalmic nerve carry magnetic navigational information? J Exp Biol 199: 1241-1244. PubMed: 9319100. 9319100 10.1242/jeb.199.5.1241

[24] Munro U, Munro JA, Phillips JB, Wiltschko R, Wiltschko W (1997) Evidence for a magnetite-based navigational 'map' in birds. Naturwissenschaften 84: 26-28. doi:10.1007/s001140050343.

[25] Wiltschko R, Stapput K, Thalau P, Wiltschko W (2010) Directional orientation of birds by the magnetic field under different light conditions. J R Soc Interface 7: S163-S177. doi:10.1098/rsif.2009.0367.focus. PubMed: 19864263. 19864263 10.1098/rsif.2009.0367.focus PMC2843996

[26] Deutschlander ME, Phillips JB, Munro U (2012) Age-Dependent Orientation to Magnetically-Simulated Geographic Displacements in Migratory Australian Silvereyes (*Zosterops l*. *lateralis*). Wilson J Ornithol 124: 467-477. doi:10.1676/11-043.1.

[27] Kishkinev D, Chernetsov N, Heyers D, Mouritsen H (2013) Migratory Reed Warblers Need Intact Trigeminal Nerves to Correct for a 1,000 km Eastward Displacement. PLOS ONE 8: e65847. doi:10.1371/journal.pone.0065847. PubMed: 23840374. 23840374 10.1371/journal.pone.0065847 PMC3694148

[28] Winklhofer M, Kirschvink JL (2010) A quantitative assessment of torque-transducer models for magnetoreception. J R Soc Interface 7: S273-S289. doi:10.1098/rsif.2009.0435.focus. PubMed: 20086054. 20086054 10.1098/rsif.2009.0435.focus PMC2843997

[29] Binhi VN (2006) Stochastic dynamics of magnetosomes and a mechanism of biological orientation in the geomagnetic field. Bioelectromagnetics 27: 58-63. doi:10.1002/bem.20178. PubMed: 16283662. 16283662 10.1002/bem.20178

[30] Johnsen S, Lohmann KJ (2005) The physics and neurobiology of magnetoreception. Nat Rev Neurosci 6: 703-712. doi:10.1038/nrn1745. PubMed: 16100517. 16100517 10.1038/nrn1745

[31] Wiltschko R, Wiltschko W (2006) Magnetoreception. Bioessays 28: 157-168. doi:10.1002/bies.20363. PubMed: 16435299. 16435299 10.1002/bies.20363

[32] Kirschvink JL, Walker MM, Diebel CE (2001) Magnetite-based magnetoreception. Curr Opin Neurobiol 11: 462-467. doi:10.1016/S0959-4388(00)00235-X. PubMed: 11502393. 11502393 10.1016/s0959-4388(00)00235-x

[33] Wiltschko W, Wiltschko R (1972) Magnetic compass of European robins. Science 176: 62-64. doi:10.1126/science.176.4030.62. PubMed: 17784420. 17784420 10.1126/science.176.4030.62

[34] Ritz T, Adem S, Schulten K (2000) A model for photoreceptor-based magnetoreception in birds. Biophys J 78: 707-718. doi:10.1016/S0006-3495(00)76629-X. PubMed: 10653784. 10653784 10.1016/S0006-3495(00)76629-X PMC1300674

[35] Rodgers CT, Hore PJ (2009) Chemical magnetoreception in birds: The radical pair mechanism. Proc Natl Acad Sci USA 106: 353-360. doi:10.1073/pnas.0711968106. PubMed: 19129499. 19129499 10.1073/pnas.0711968106 PMC2626707

[36] Solovýov IA, Hore PJ, Ritz T, Schulten K (2011) A chemical compass for bird navigation. Quantum Effects in Biology. New York City: Cambridge University Press.

[37] Henbest KB, Kukura P, Rodgers CT, Hore PJ, Timmel CR (2004) Radio frequency magnetic field effects on a radical recombination reaction: a diagnostic test for the radical pair mechanism. J Am Chem Soc 126: 8102-8103. doi:10.1021/ja048220q. PubMed: 15225036. 15225036 10.1021/ja048220q

[38] Ritz T, Thalau P, Phillips JB, Wiltschko R, Wiltschko W (2004) Resonance effects indicate a radical-pair mechanism for avian magnetic compass. Nature 429: 177-180. doi:10.1038/nature02534. PubMed: 15141211. 15141211 10.1038/nature02534

[39] Ritz T, Wiltschko R, Hore PJ, Rodgers CT, Stapput K et al. (2009) Magnetic compass of birds is based on a molecule with optimal directional sensitivity. Biophys J 96: 3451-3457. doi:10.1016/j.bpj.2008.11.072. PubMed: 19383488. 19383488 10.1016/j.bpj.2008.11.072 PMC2718301

[40] Thalau P, Ritz T, Stapput K, Wiltschko R, Wiltschko W (2005) Magnetic compass orientation of migratory birds in the presence of a 1.315 MHz oscillating field. Naturwissenschaften 92: 86-90. doi:10.1007/s00114-004-0595-8. PubMed: 15614508. 15614508 10.1007/s00114-004-0595-8

[41] Lau JC, Rodgers CT, Hore PJ (2012) Compass magnetoreception in birds arising from photo-induced radical pairs in rotationally disordered cryptochromes. J R Soc Interface, 9: 3329–37. PubMed: 22977104. 22977104 10.1098/rsif.2012.0374 PMC3481564

[42] Solov’yov IA, Domratcheva T, Moughal Shahi AR, Schulten K (2012) Decrypting Cryptochrome: Revealing the Molecular Identity of the Photoactivation Reaction. J Am Chem Soc. 10.1021/ja3074819 PMC3500783 23009093

[43] Burger T, Lucová M, Moritz RE, Oelschläger HH, Druga R et al. (2010) Changing and shielded magnetic fields suppress c-Fos expression in the navigation circuit: input from the magnetosensory system contributes to the internal representation of space in a subterranean rodent. J R Soc Interface 7: 1275-1292. doi:10.1098/rsif.2009.0551. PubMed: 20219838. 20219838 10.1098/rsif.2009.0551 PMC2894883

[44] Muheim R, Bäckman J, Akesson S (2002) Magnetic compass orientation in European robins is dependent on both wavelength and intensity of light. J Exp Biol 205: 3845-3856. PubMed: 12432008. 12432008 10.1242/jeb.205.24.3845

[45] Bingman VP (1987) Earth’s magnetism and the nocturnal orientation of migratory European robins. Auk 104: 523-525. doi:10.2307/4087555.

[46] Wiltschko W, Wiltschko R (1975) The interaction of stars and magnetic field in the orientation system of night migrating birds. I. Autumn experiments with European Warblers (gen. *Sylvia*). Z Tierpsychol 37: 337-355. PubMed: 1229769. 1229769 10.1111/j.1439-0310.1975.tb00885.x

[47] Wahlsten D, Bachmanov A, Finn DA, Crabbe JC (2006) Stability of inbred mouse strain differences in behavior and brain size between laboratories and across decades. Proc Natl Acad Sci USA 103: 16364-16369. doi:10.1073/pnas.0605342103. PubMed: 17053075. 17053075 10.1073/pnas.0605342103 PMC1618164

[48] Iivonen H, Nurminen L, Harri M, Tanila H, Puoliväli J (2003) Hypothermia in mice tested in Morris water maze. Behav Brain Res 141: 207-213. doi:10.1016/S0166-4328(02)00369-8. PubMed: 12742257. 12742257 10.1016/s0166-4328(02)00369-8

[49] Merritt R, Purcell C, Stroink G (1983) Uniform magnetic field produced by three, four, and five square coils. Rev Sci Instrum 54: 879-882. doi:10.1063/1.1137480.

[50] Phillips JB (1986) Magnetic compass orientation in the Eastern red-spotted newt (*Notophthalmus viridescens*). J Comp Physiol A 158: 103-109. doi:10.1007/BF00614524. PubMed: 3723427. 3723427 10.1007/BF00614524

[51] Kirschvink JL (1992) Uniform magnetic fields and double-wrapped coil systems: Improved techniques for the design of bioelectromagnetic experiments. Bioelectromagnetics 13: 401-411. doi:10.1002/bem.2250130507. PubMed: 1445421. 1445421 10.1002/bem.2250130507

[52] Batschelet E (1981) Circular statistics in biology. New York: Academic Press.

[53] Upchurch M, Wehner JM (1988) Differences between inbred strains of mice in Morris water maze performance. Behav Genet 18: 55-68. doi:10.1007/BF01067075. PubMed: 3365197. 3365197 10.1007/BF01067075

[54] Calhoun ME, Kurth D, Phinney AL, Long JM, Hengemihle J et al. (1998) Hippocampal neuron and synaptophysin-positive bouton number in aging C57BL/6 mice. Neurobiol Aging 19: 599-606. doi:10.1016/S0197-4580(98)00098-0. PubMed: 10192220. 10192220 10.1016/s0197-4580(98)00098-0

[55] Phillips JB, Muheim R, Jorge PE (2010) A behavioral perspective on the biophysics of the light-dependent magnetic compass: a link between directional and spatial perception? J Exp Biol 213: 3247–3255. doi:10.1242/jeb.020792. PubMed: 20833916. 20833916 10.1242/jeb.020792

[56] Derdikman D, Moser EI (2010) A manifold of spatial maps in the brain. Trends Cogn Sci 14: 561-569. doi:10.1016/j.tics.2010.09.004. PubMed: 20951631. 20951631 10.1016/j.tics.2010.09.004

[57] Dudchenko PA, Davidson M (2002) Rats use a sense of direction to alternate on T-mazes located in adjacent rooms. Anim Cogn 5: 115-118. doi:10.1007/s10071-002-0134-y. PubMed: 12150036. 12150036 10.1007/s10071-002-0134-y

[58] Stackman RW, Golob EJ, Bassett JP, Taube JS (2003) Passive Transport Disrupts Directional Path Integration by Rat Head Direction Cells. J Neurophysiol 90: 2862-2874. doi:10.1152/jn.00346.2003. PubMed: 12890795. 12890795 10.1152/jn.00346.2003

[59] Sharp PE (2006) Subicular place cells generate the same “map” for different environments: Comparison with hippocampal cells. Behav Brain Res 174: 206-214. doi:10.1016/j.bbr.2006.05.034. PubMed: 16859764. 16859764 10.1016/j.bbr.2006.05.034

[60] Brown JE, Yates BJ, Taube JS (2002) Does the vestibular system contribute to head direction cell activity in the rat? Physiol Behav 77: 743-748. doi:10.1016/S0031-9384(02)00928-9. PubMed: 12527029. 12527029 10.1016/s0031-9384(02)00928-9

[61] Knierim JJ, Kudrimoti HS, McNaughton BL (1995) Place cells, head direction cells, and the learning of landmark stability. J Neurosci 15: 1648–1659. PubMed: 7891125. 7891125 10.1523/JNEUROSCI.15-03-01648.1995 PMC6578145

[62] Stackman RW, Clark AS, Taube JS (2002) Hippocampal spatial representations require vestibular input. Hippocampus 12: 291-303. doi:10.1002/hipo.1112. PubMed: 12099481. 12099481 10.1002/hipo.1112 PMC1823522

[63] Stackman RW, Taube JS (1998) Firing properties of rat lateral mammillary single units: head direction, head pitch, and angular head velocity. J Neurosci 18: 9020-9037. PubMed: 9787007. 9787007 10.1523/JNEUROSCI.18-21-09020.1998 PMC1550347

[64] Semm P, Nohr D, Demaine C, Wiltschko W (1984) Neural basis of the magnetic compass: interactions of visual, magnetic and vestibular inputs in the pigeon’s brain. J Comp Physiol A Sens Neural Behav Physiol 155: 283-288. doi:10.1007/BF00610581.

[65] Wu LQ, Dickman JD (2012) Neural correlates of a magnetic sense. Sci Signal 336: 1054. 10.1126/science.1216567 22539554

[66] Wu LQ, Dickman JD (2011) Magnetoreception in an Avian Brain in Part Mediated by Inner Ear Lagena. Curr Biol 21: 418-423. doi:10.1016/j.cub.2011.01.058. PubMed: 21353559. 21353559 10.1016/j.cub.2011.01.058 PMC3062271

[67] Lauwers M, Pichler P, Edelman NB, Resch GP, Ushakova L et al. (2013) An Iron-Rich Organelle in the Cuticular Plate of Avian Hair Cells. Curr Biol CB, 23: 924–9. PubMed: 23623555. 23623555 10.1016/j.cub.2013.04.025

[68] Cheung A, Zhang S, Stricker C, Srinivasan MV (2008) Animal navigation: general properties of directed walks. Biol Cybern 99: 197-217. doi:10.1007/s00422-008-0251-z. PubMed: 18781320. 18781320 10.1007/s00422-008-0251-z

[69] Cheung A, Zhang S, Stricker C, Srinivasan MV (2007) Animal navigation: the difficulty of moving in a straight line. Biol Cybern 97: 47-61. doi:10.1007/s00422-007-0158-0. PubMed: 17520273. 17520273 10.1007/s00422-007-0158-0

[70] Diego-Rasilla FJ, Luengo RM, Phillips JB (2010) Light-dependent magnetic compass in Iberian green frog tadpoles. Naturwissenschaften 97: 1077-1088. doi:10.1007/s00114-010-0730-7. PubMed: 20978882. 20978882 10.1007/s00114-010-0730-7

[71] Mouritsen H, Hore PJ (2012) The magnetic retina: light-dependent and trigeminal magnetoreception in migratory birds. Curr Opin Neurobiol 22: 343-352. doi:10.1016/j.conb.2012.01.005. PubMed: 22465538. 22465538 10.1016/j.conb.2012.01.005

[72] Olcese J, Reuss S, Vollrath L (1985) Evidence for the involvement of the visual system in mediating magnetic field effects on pineal melatonin synthesis in the rat. Brain Res 333: 382-384. doi:10.1016/0006-8993(85)91598-7. PubMed: 3995304. 3995304 10.1016/0006-8993(85)91598-7

[73] Olcese J, Reuss S, Stehle J, Steinlechner S, Vollrath L (1988) Responses of the mammalian retina to experimental alteration of the ambient magnetic field. Brain Res 448: 325-330. doi:10.1016/0006-8993(88)91271-1. PubMed: 3378153. 3378153 10.1016/0006-8993(88)91271-1

[74] Möller A, Sagasser S, Wiltschko W, Schierwater B (2004) Retinal cryptochrome in a migratory passerine bird: a possible transducer for the avian magnetic compass. Naturwissenschaften 91: 585-588. doi:10.1007/s00114-004-0578-9. PubMed: 15551029. 15551029 10.1007/s00114-004-0578-9

[75] Niessner C, Denzau S, Gross JC, Peichl L, Bischof HJ et al. (2011) Avian Ultraviolet/Violet cones identified as probable magnetoreceptors. PLOS ONE 6: e20091 PubMed: 21647441. 21647441 10.1371/journal.pone.0020091 PMC3102070

[76] Tryon VL, Kim EU, Zafar TJ, Unruh AM, Staley SR et al. (2012) Magnetic field polarity fails to influence the directional signal carried by the head direction cell network and the behavior of rats in a task requiring magnetic field orientation. Behav Neurosci 126: 835–844. doi:10.1037/a0030248. PubMed: 23025828. 23025828 10.1037/a0030248

[77] Ritz D (1991) Polarised light responses in the shrimp *Palaemonetes vulgaris* (Say). J Exp Mar Biol Ecol 154: 245-250. doi:10.1016/0022-0981(91)90167-U.

[78] Vácha M, Puzová T, Kvícalová M (2009) Radio frequency magnetic fields disrupt magnetoreception in American cockroach. J Exp Biol 212: 3473–3477. doi:10.1242/jeb.028670. PubMed: 19837889. 19837889 10.1242/jeb.028670

[79] Gauger EM, Rieper E, Morton JJL, Benjamin SC, Vedral V (2011) Sustained quantum coherence and entanglement in the avian compass. Phys Rev Lett 106: 40503. doi:10.1103/PhysRevLett.106.040503. PubMed: 21405313. 10.1103/PhysRevLett.106.040503 21405313

