# Supplementary figures and images for: Rapid Learning of Magnetic Compass Direction by C57BL/6 Mice in a 4-Armed ‘Plus’ Water Maze
Source: PLoS One. 2013 Aug 30;8(8):e73112. doi: 10.1371/journal.pone.0073112 (PMC3758273; doi:10.1371/journal.pone.0073112)

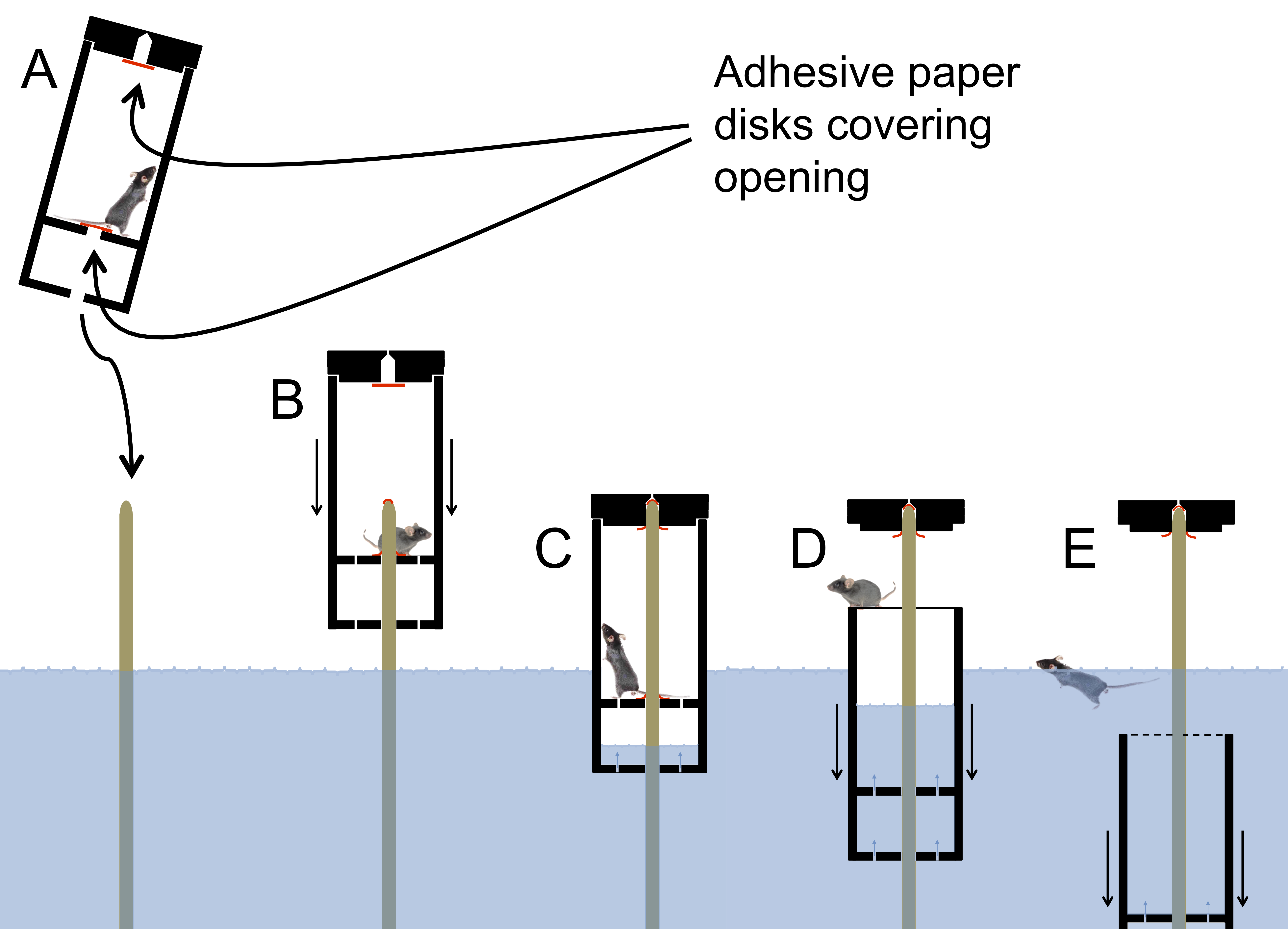

Supplement: Figure S1 — Water maze release device. A) Mouse was transported from the holding shelf to the testing arena inside the opaque release device. Disks of paper towel attached with a ring cut from a self-adhesive label covered openings in both the top and bottom of the chamber. B) The release device was then slid slowly down onto a vertical Plexiglas rod extending up above the water in the center of the plus maze. The top of the rod inserted firmly into a socket centered on the bottom of the lid. C) The buoyancy of the lower section of the release device and the rate of inflow of water through four small openings were adjusted so that once the release device was in place, the lower section remained snugly up against the lid long enough for the observer to quietly exit the testing room and close the intervening door without being observed. D) Water slowly entered the lower chamber, and eventually the upper chamber, through the 4 small holes, causing the lower section of the release device to gradually separate from the lid and sink lower in the water. As the lower section filled with water and separated from the lid, the mouse invariably crawled up onto the top rim of the cylinder and spent 20-35 sec walking around the rim looking in all directions. E) The lower section of the release device gradually submerged, taking 45-50 sec for the top rim to sink below the surface, forcing the mouse to swim. (TIFF) [file pone.0073112.s001.tiff]

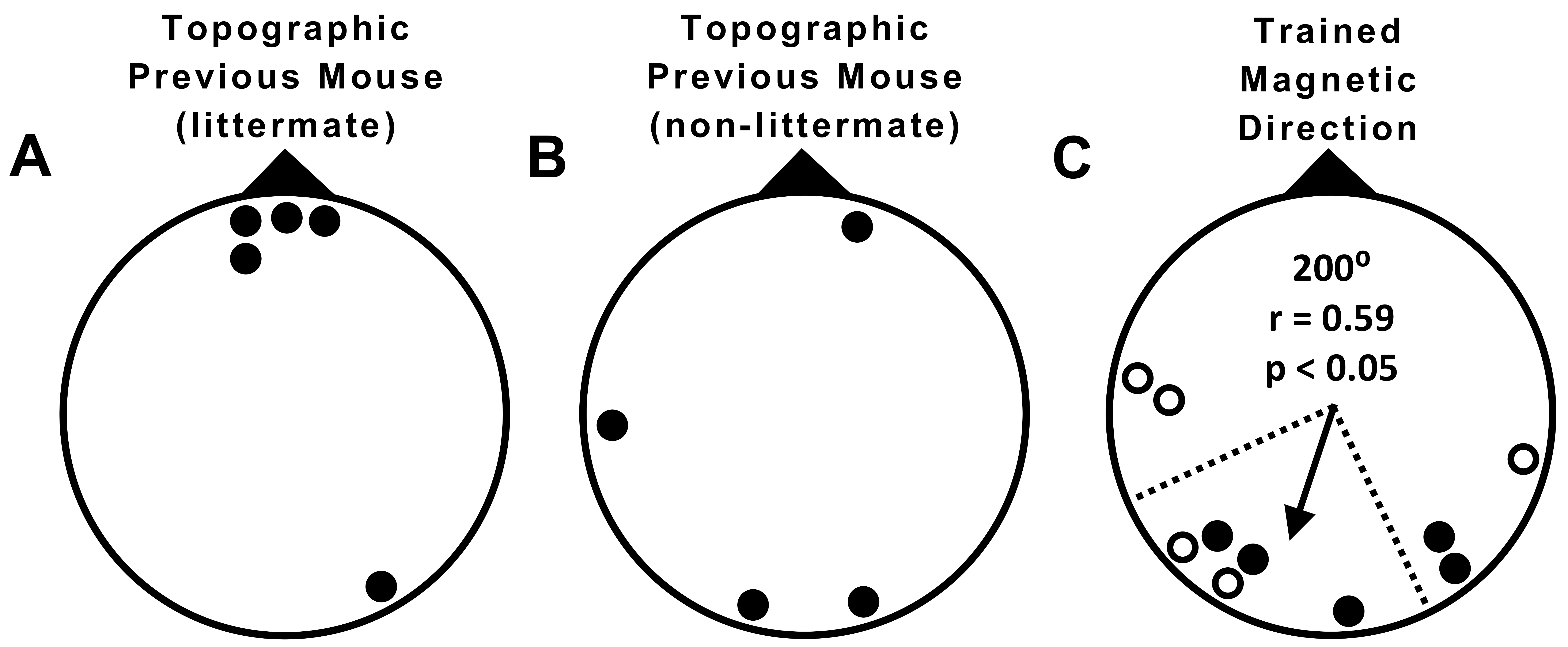

Supplement: Figure S3 — Evidence for olfactory following. Example of data obtained in some tests before a cleaning protocol was introduced between testing trials to disperse directional olfactory cues left by the previous mouse. Distributions of bearings in A & B show the deviations between the topographic bearings of pairs of mice tested sequentially that were either littermates (A), or non-littermates (B); black arrow indicates the bearing of the first mouse of the pair. Although the alignment of the magnetic field was rotated by 90° between trials, A) the topographic bearings of 4 of 5 mice tested immediately after a littermate exhibited a topographic bearing that differed from that of the previous mouse by less than 20°. In contrast, B) the topographic bearings of the 4 mice tested immediately after a non-littermate showed no evidence of clustering. C) The deviations of the magnetic bearings of all 10 mice relative to the trained magnetic direction were non-randomly distributed, and the 95% confidence intervals included the direction opposite the trained direction (solid symbols—bearings from mice tested after a non-littermate, open symbols—bearings from mice tested after a littermate). The water temperature in testing was 31 °C, which causes a reversal in the direction of orientation relative to the trained magnetic direction (see Materials & Methods). (TIFF) [file pone.0073112.s003.tiff]

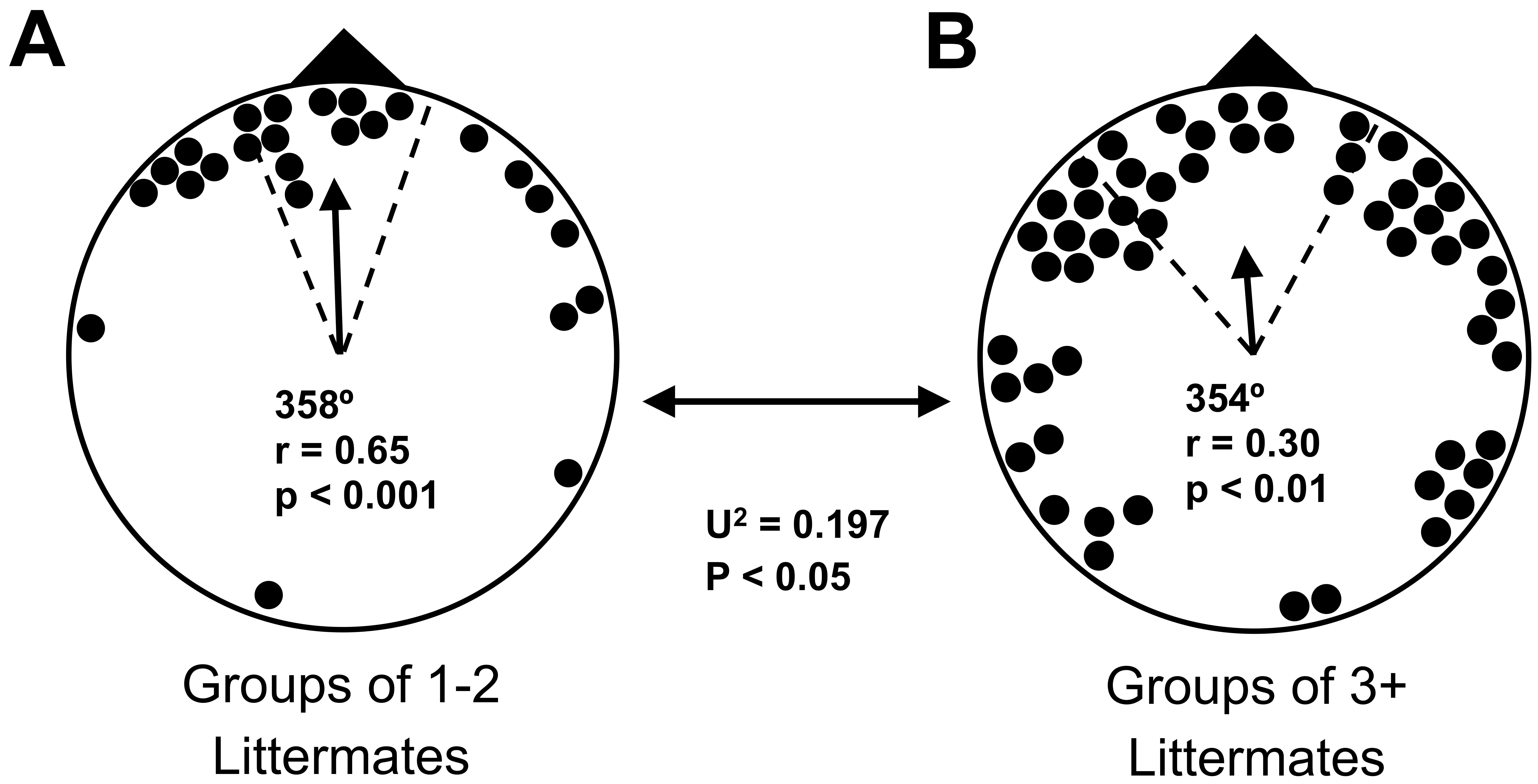

Supplement: Figure S4 — Number of littermates in the same testing group may affect the consistency of orientation. Although mice from littermate groups of both (A) 1-2 and (B) 3+ exhibited significant orientation in the trained magnetic direction (p < 0.01, Rayleigh test), mice in littermate groups of 1-2 to exhibited less scatter (p < 0.05, Watson U2 test). Each mouse was trained in one of four directions (submerged platform towards magnetic north, east, south or west), and then tested in one of four alignments of the magnetic field (magnetic north aligned towards geomagnetic North, East, South, or West). Bearings are plotted relative to trained magnetic direction (black triangle at the top of each diagram). Arrows at the center of each distribution show the mean vector bearing; the length of the arrow is proportional to the mean vector length (‘r’) with the radius of the circle corresponding to r = 1. Dashed lines show the 95% C.I. for the mean vector bearing. (TIFF) [file pone.0073112.s004.tiff]
