# Supplementary material for: Rapid Learning of Magnetic Compass Direction by C57BL/6 Mice in a 4-Armed ‘Plus’ Water Maze
Source: PLoS One. 2013 Aug 30;8(8):e73112. doi: 10.1371/journal.pone.0073112 (PMC3758273; doi:10.1371/journal.pone.0073112)
Supplement: Table S1 — Responses of south trained mice in Figure S3. (DOCX) [file pone.0073112.s005.docx]

Table S1. Responses of south trained mice in Fig S3.

| **Mouse Testing Sequence** | **Testing Field** | **Topographic**  **Bearing (°)** | **Magnetic**  **Bearing (°)** | **Bearing Relative to Trained**  **Direction (°)** | **Deviation**  **Topographic Bearings (°) of Littermates** | **Deviation Topographic Bearings (°) of**  **Non-littermates** |
| --- | --- | --- | --- | --- | --- | --- |
| 1 | W | 230 | 320 | 140 | ------ | ------ |
| 2 | S | 211 | 31 | 211 | 349 | ------ |
| 3 | E | 48 | 318 | 138 | ------ | 197 |
| 4 | N | 48 | 48 | 228 | 360 | ------ |
| 5 | W | 315 | 45 | 225 | ------ | 267 |
| 6 | S | 105 | 285 | 105 | 150 | ------ |
| 7 | E | 119 | 29 | 209 | ------ | 14 |
| 8 | N | 102 | 102 | 282 | 343 | ------ |
| 9 | W | 265 | 355 | 175 | ------ | 163 |
| 10 | S | 276 | 96 | 276 | 11 | ------ |
