# Supplementary material for: Rapid Learning of Magnetic Compass Direction by C57BL/6 Mice in a 4-Armed ‘Plus’ Water Maze
Source: PLoS One. 2013 Aug 30;8(8):e73112. doi: 10.1371/journal.pone.0073112 (PMC3758273; doi:10.1371/journal.pone.0073112)
Supplement: Table S2 — Responses included in Figure 2 †. (DOCX) [file pone.0073112.s006.docx]

Table S2—Responses included in Fig 2^†^.

| **Group** | **Test Number** | **Testing Order^‡^** | **Testing Field** | **Size of Littermate Group** | **Bearing Relative to Topographic North (°)** | **Bearing Relative to Magnetic North (°)** | **Bearing Relative to Trained Magnetic Direction (°)** |
| --- | --- | --- | --- | --- | --- | --- | --- |
| **West Trained** | 1 | 1 | S | 3 | 174 | 354 | 84 |
|  |  | 2 | E | 1 | 342 | 252 | 342 |
|  |  | 4 | W | 3 | 242 | 332 | 62 |
| **East Trained** | 2 | 1 | N | 4 | 216 | 216 | 126 |
|  |  | 2 | W | 1 | 348 | 78 | 348 |
|  |  | 3 | S | 4 | 313 | 133 | 43 |
| **West Trained** | 3 | 1 | W | 3 | 161 | 251 | 341 |
|  |  | 2 | S | 2 | 142 | 322 | 52 |
|  |  | 3 | E | 3 | 294 | 204 | 294 |
|  |  | 4 | N | 3 | 322 | 322 | 52 |
| **South Trained** | 4 | 1 | S | 3 | 110 | 290 | 110 |
|  |  | 2 | E | 3 | 292 | 202 | 22 |
|  |  | 3 | N | 3 | 70 | 90 | 270 |
|  |  | 4 | W | 3 | 57 | 147 | 327 |
| **North Trained** | 5 | 1 | E | 5 | 161 | 71 | 71 |
|  |  | 3 | W | 5 | 272 | 2 | 2 |
| **East Trained** | 6 | 1 | N | 2 | 84 | 84 | 354 |
|  |  | 2 | W | 2 | 343 | 73 | 343 |
|  |  | 3 | S | 3 | 269 | 89 | 359 |
|  |  | 4 | E | 2 | 17 | 287 | 197 |

†Four mice excluded (see Materials and Methods). ^‡^Order of rows corresponds to sequence in which mice were trained on the day prior to testing.
