# Supplementary material for: Rapid Learning of Magnetic Compass Direction by C57BL/6 Mice in a 4-Armed ‘Plus’ Water Maze
Source: PLoS One. 2013 Aug 30;8(8):e73112. doi: 10.1371/journal.pone.0073112 (PMC3758273; doi:10.1371/journal.pone.0073112)
Supplement: Table S3 — Responses included in Figure 3††. (DOCX) [file pone.0073112.s007.docx]

Table S3. Responses included in Fig 3**^††^**.

| **Group** | **Test Number** | **Testing Order** | **Testing Field** | **Size of Littermate Group** | **Bearing Relative to Topographic North (°)** | **Bearing Relative to Magnetic North (°)** | **Bearing Relative to Trained Magnetic Direction (°)** |
| --- | --- | --- | --- | --- | --- | --- | --- |
| **North Trained** | 1 | 3 | W | 2 | 223 | 323 | 323 |
|  |  | 1 | S | 2 | 298 | 118 | 118 |
|  |  | 2 | N | 1 | 11 | 11 | 11 |
| **South Trained** | 2 | 3 | N | 2 | 164 | 164 | 344 |
|  |  | 2 | W | 2 | 168 | 258 | 78 |
|  |  | 1 | E | 3 | 314 | 224 | 44 |
| **South Trained** | 3 | 2 | W | 2 | 69 | 169 | 339 |
|  |  | 4 | S | 2 | 44 | 224 | 44 |
|  |  | 3 | N | 1 | 96 | 96 | 276 |
|  |  | 1 | E | 3 | 28 | 298 | 118 |
| **South Trained** | 4 | 2 | W | 2 | 56 | 146 | 326 |
|  |  | 3 | S | 4 | 305 | 125 | 305 |
|  |  | 1 | E | 4 | 228 | 138 | 318 |
|  |  | 4 | N | 4 | 41 | 41 | 221 |
| **South Trained** | 5 | 4 | E | 2 | 227 | 137 | 317 |
|  |  | 3 | W | 3 | 57 | 147 | 327 |
|  |  | 1 | S | 3 | 247 | 67 | 247 |
| **North Trained** | 6 | 4 | S | 1 | 263 | 83 | 83 |
|  |  | 1 | W | 5 | 126 | 216 | 216 |
|  |  | 3 | N | 5 | 5 | 5 | 5 |
| **South Trained** | 7 | 2 | N | 2 | 211 | 211 | 31 |
|  |  | 4 | E | 4 | 349 | 259 | 79 |
|  |  | 3 | W | 4 | 218 | 308 | 128 |
|  |  | 1 | S | 4 | 320 | 140 | 320 |
| **South Trained** | 8 | 1 | W | 2 | 91 | 181 | 1 |
|  |  | 4 | S | 2 | 315 | 135 | 315 |
|  |  | 3 | N | 3 | 314 | 314 | 134 |
| **North Trained** | 9 | 3 | W | 6 | 229 | 319 | 319 |
|  |  | 2 | S | 6 | 353 | 173 | 173 |
|  |  | 1 | N | 6 | 346 | 346 | 346 |
|  |  | 4 | E | 6 | 114 | 24 | 24 |
| **North Trained** | 10 | 3 | N | 3 | 51 | 51 | 51 |
|  |  | 1 | W | 3 | 360 | 90 | 90 |
| **South Trained** | 11 | 2 | S | 2 | 337 | 157 | 337 |
|  |  | 1 | N | 4 | 127 | 127 | 307 |
| **South Trained** | 12 | 4 | S | 2 | 53 | 233 | 53 |
|  |  | 1 | W | 2 | 90 | 180 | 0 |
|  |  | 3 | E | 3 | 295 | 205 | 25 |
|  |  | 2 | N | 3 | 82 | 82 | 262 |
| **South Trained** | 13 | 3 | S | 2 | 309 | 129 | 309 |
|  |  | 1 | W | 5 | 27 | 117 | 297 |
|  |  | 4 | N | 5 | 68 | 68 | 248 |
|  |  | 2 | E | 5 | 235 | 145 | 325 |
| **South Trained** | 14 | 4 | W | 2 | 94 | 184 | 4 |
| **South Trained** | 15 | 1 | S | 5 | 332 | 152 | 332 |
|  |  | 3 | E | 5 | 227 | 137 | 317 |
|  |  | 2 | N | 5 | 230 | 230 | 50 |
| **South Trained** | 16 | 2 | S | 3 | 262 | 82 | 262 |
|  |  | 4 | E | 3 | 141 | 51 | 231 |
|  |  | 1 | N | 3 | 242 | 242 | 62 |
|  |  | 3 | W | 3 | 236 | 344 | 164 |
| **South Trained** | 17 | 2 | N | 3 | 161 | 161 | 341 |
|  |  | 3 | W | 3 | 356 | 86 | 266 |
|  |  | 1 | S | 3 | 218 | 38 | 218 |
| **South Trained** | 18 | 3 | N | 4 | 213 | 213 | 33 |
|  |  | 2 | W | 3 | 86 | 176 | 356 |
| **South Trained** | 19 | 3 | E | 3 | 222 | 132 | 312 |
|  |  | 1 | N | 3 | 223 | 223 | 43 |
|  |  | 4 | W | 4 | 29 | 119 | 299 |
|  |  | 2 | S | 4 | 116 | 296 | 116 |

**††**Sixteen mice were excluded (see text), 7 of these because the mouse’s cage bumped the ‘vestibule’ as it was removed from the holding shelf. Vestibule was added prior to these experiments to provide electromagnetic shielding of holding shelves (Fig S2).
